# Programmable Tetrahedral DNA‐RNA Nanocages Woven with Stimuli‐Responsive siRNA for Enhancing Therapeutic Efficacy of Multidrug‐Resistant Tumors

**DOI:** 10.1002/advs.202404112

**Published:** 2024-06-25

**Authors:** Changmai Chen, Maocheng Yu, Qing Li, Ying Zhou, Mengting Zhang, Shanyu Cai, Jiaojiao Yu, Zhongnan Huang, Jiaan Liu, Ye Kuang, Xinjing Tang, Wei Chen

**Affiliations:** ^1^ Fujian Key Laboratory of Drug Target Discovery and Structural and Functional Research, School of Pharmacy Fujian Medical University Fuzhou 350122 China; ^2^ State Key Laboratory of Natural and Biomimetic Drugs School of Pharmaceutical Sciences Peking University Beijing 100191 China

**Keywords:** chemotherapy, multidrug resistance, RNA interference, tetrahedral DNA‐RNA nanocages

## Abstract

Multidrug resistance (MDR) is a major obstacle limiting the effectiveness of chemotherapy against cancer. The combination strategy of chemotherapeutic agents and siRNA targeting drug efflux has emerged as an effective cancer treatment to overcome MDR. Herein, stimuli‐responsive programmable tetrahedral DNA‐RNA nanocages (TDRN) have been rationally designed and developed for dynamic co‐delivery of the chemotherapeutic drug doxorubicin and P‐glycoprotein (P‐gp) siRNA. Specifically, the sense and antisense strand sequences of the P‐gp siRNA, which are programmable bricks with terminal disulfide bond conjugation, are precisely embedded in one edge of the DNA tetrahedron. TDRN provides a stimuli‐responsive release element for dynamic control of functional cargo P‐gp siRNA that is significantly more stable than the “tail‐like” TDN nanostructures. The stable and highly rigid 3D nanostructure of the siRNA‐organized TDRN nanocages demonstrated a notable improvement in the stability of RNase A and mouse serum, as well as long‐term storage stability for up to 4 weeks, as evidenced by this study. These biocompatible and multifunctional TDRN nanocarriers with gold nanocluster‐assisted delivery (TDRN@Dox@AuNC_p_) are successfully used to achieve synergistic RNAi/Chemo‐therapy in vitro and in vivo. This programmable TDRN drug delivery system, which integrates RNAi therapy and chemotherapy, offers a promising approach for treating multidrug‐resistant tumors.

## Graphical abstract

A programmable tetrahedral DNA‐RNA nanocage (TDRN) woven with stimuli‐responsive siRNA is rationally designed for dynamic co‐delivery of siRNA and chemotherapeutics for enhancing therapeutic efficacy of multidrug‐resistant tumors. The programmable TDRN nanocages significantly improve RNase A, long‐term storage, and mouse serum stability, and demonstrate synergistic RNAi/Chemo‐therapy in vitro and in vivo, offering a promising strategy to overcome MDR in cancer treatment.

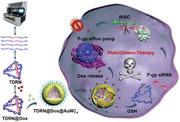

## 1 Introduction

Multidrug resistance (MDR) remains a critical issue for effective chemotherapy against cancer.^[^ ^1^ ^]^ P‐glycoprotein (P‐gp), a major drug‐efflux pump, is involved in the transport of chemotherapeutic drugs out of MDR cancer cells. A rapidly growing number of studies have focused on combining chemotherapy with RNA interference (RNAi) therapy targeting the P‐gp efflux pump to overcome multidrug resistance. However, there are still many challenges to siRNA delivery that need to be addressed, including susceptibility to nuclease degradation,^[^ ^2^ ^]^ inefficient cellular uptake,^[^ ^3^ ^]^ and failure to escape from the lysosome.^[^ ^4^ ^]^ A wide variety of nano‐based drug delivery systems have been designed for RNAi therapy,^[^ ^5^ ^]^ including liposomes, polymers, dendrimers, inorganic nanomaterials, etc. Recently, oligoarginine (CR9) cationic gold nanoclusters (AuNC@CR9) with sizes smaller than 3 nm by a simple one‐step reaction were developed for efficient delivery of siRNA and aptamers to enhance the cellular uptake and gene silencing.^[^ ^6^ ^]^ The potential toxicity of these nanocarriers and the lack of controllable release in response to stimuli have partly limited their widespread use in cancer treatment.^[^ ^7^ ^]^

DNA nanostructures with controlled size and shape were widely employed in drug delivery systems due to their programmable assembly, excellent biocompatibility, and high biophysical controllability. Tetrahedral DNA nanostructures (TDN) have attracted considerable attention due to their exceptional addressability,^[^ ^8^ ^]^ programmability,^[^ ^9^ ^]^ and remarkable penetration.^[^ ^10^ ^]^ Various studies have shown that TDN is highly capable of loading a series of cargoes, including small molecule drugs,^[^ ^11^ ^]^ functional oligonucleotides,^[^ ^12^ ^]^ peptides,^[^ ^13^ ^]^ proteins, and enzymes.^[^ ^14^ ^]^ Therefore, we hypothesized that TDN is a promising candidate for the co‐delivery of siRNA and chemotherapeutic drugs into MDR tumor cells for synergistic therapy.

Previous reports have mainly focused on the “tail‐like” TDN strategy via sticky ends to deliver siRNA, miRNA, antisense oligonucleotides (ASO), aptamers, CPG, etc.^[^ ^15^ ^]^ However, these “tail‐like” TDN nanostructures, which are easy and convenient to prepare, serve only as static devices for holding functional cargo oligonucleotides, but lack dynamic, stimuli‐responsive release elements.^[^ ^16^ ^]^ The cargo oligonucleotides of the static TDN nanocarriers with sticky ends are susceptible to degradation by nucleases in complex living systems.^[^ ^17^ ^]^ Recently, researchers have increasingly emphasized the original stable structure of the TDN scaffold itself. The native cavity of TDN for loading cargo drugs has been validated and used to encapsulate melittin,^[^ ^18^ ^]^ gold nanoparticles,^[^ ^19^ ^]^ leading to major advances in the dynamic delivery of functional nucleic acids, including the encapsulation of siRNA^[^ ^16a^ ^]^ and ASO^[^ ^20^ ^]^ into the space within the TDN scaffold for effective and stable cargo delivery.

TDN nanocage strategies will demonstrate promising applications for studying the spatial modulation of functional genes and stimuli‐responsive nucleic acid prodrugs.^[^ ^21^ ^]^ However, there are still few studies based on the spatial control of functional siRNA that are organized in the edge of the DNA tetrahedron. Inspired by the self‐assembled caged siRNA nanoparticles shown in our previous studies,^[^ ^22^ ^]^ we designed a stimuli‐sensitive tetrahedral DNA‐RNA nanocage (TDRN) for the dynamic co‐delivery of P‐gp siRNA and the chemotherapeutic agent doxorubicin (Dox, also known as adriamycin, ADR). Stimuli‐responsive cleavage of the disulfide bonds enables tetrahedral DNA‐RNA nanocages to open the stable and highly rigid nanostructure, facilitating the release of the active P‐gp siRNA cargo to circumvent multidrug resistance. Furthermore, considering that TDN mainly enters lysosomes after cellular endocytosis,^[^ ^23^ ^]^ we synthesized a non‐cytotoxic polyethyleneimine (PEI) cationic gold nanocluster (AuNC@PEI) to facilitate the delivery of TDRN.

**Scheme 1:**
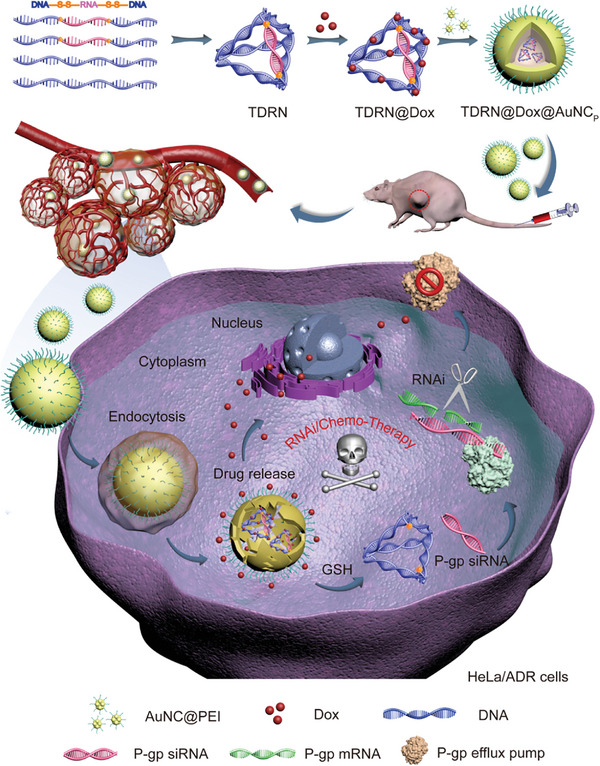
Schematic illustration of programmable tetrahedral DNA‐RNA nanocages woven with stimuli‐responsive siRNA (TDRN@Dox@AuNC_p_) for synergistic RNAi/Chemo‐therapy of multidrug‐resistant tumors. ‐SS‐, disulfide bond; Dox, Doxorubicin; P‐gp, P‐glycoprotein; AuNC_p_, PEI modified gold nanoclusters.

Herein, we first constructed a programmable tetrahedral DNA‐RNA nanocage woven with stimuli‐responsive siRNA, which was employed as a dynamic co‐delivery nanocarrier for P‐gp siRNA and Dox into MDR tumor cells, protecting the loaded cargos by benefiting from the rigid structure of the TDRN (**Scheme** **1**). Unlike the previously reported “tail‐like” TDN strategy using sticky ends, these novel programmable TDRN were incorporated with stimuli‐responsive, cleavable disulfide bonds of siRNA that respond to the cellular reducing environment. To improve RNase A and long‐term storage stability and mouse serum stability, we have rationally embedded the siRNA into one edge of the TDRN, resulting in increased resistance to RNase A degradation compared to naked siRNA. The reduced mRNA and protein expression levels of P‐gp were evaluated in Dox‐resistance HeLa cells (HeLa/ADR) and established multidrug‐resistant HeLa/ADR tumors. The biocompatible and multifunctional TDRN@Dox@AuNC_p_ nanocarriers can effectively kill MDR tumor cells and show a dramatically enhanced synergistic anti‐tumor effect in vitro and in vivo. This innovative approach holds great potential for overcoming drug resistance and improving the efficacy of chemotherapy in cancer treatment.

## 2 Results and Discussion

### 2.1 Design and Characterization of TDRN and TDRN@Dox@AuNC_p_

**Figure 1:**
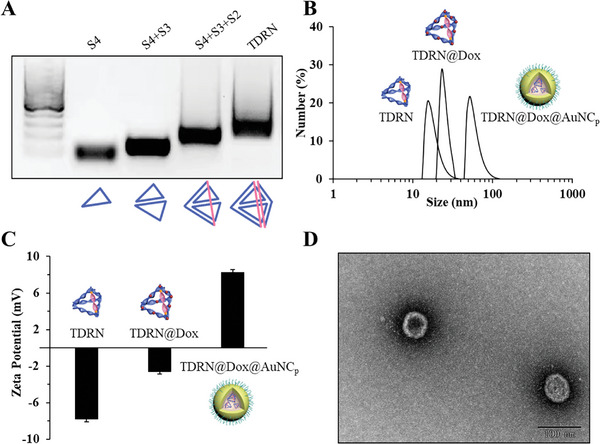
Characterization of TDRN, TDRN@Dox and TDRN@Dox@AuNC_p_. A) 1.5% agarose gel electrophoresis confirming the synthesis of TDRN. B) DLS characterization of TDRN (PDI, 0.221), TDRN@Dox (PDI, 0.239), and TDRN@Dox@AuNC_p_ (PDI, 0.184). C) Zeta potentials of TDRN, TDRN@Dox, and TDRN@Dox@AuNC_p_. D) TEM characterization of TDRN@Dox@AuNC_p_.

The phosphoramidites of thiol‐modified C6 S‐S (SS), 19‐nt RNA nucleotides (RNA), the phosphoramidites of thiol‐modified C6 S‐S (SS), and 15‐nt DNA nucleotides (DNA) were sequentially coupled to 38‐nt DNA nucleotides (DNA) using a LK‐48E DNA/RNA solid‐phase synthesizer. DNA‐SS‐RNA‐SS‐DNA oligonucleotides targeting P‐gp with two disulfide bond modifications were designed and synthesized (Table S1 and Figure S1, Supporting Information). All oligonucleotides used in this study were purified by HPLC and characterized by ESI‐MS (see the Supporting Information.). We subsequently constructed glutathione (GSH)‐responsive tetrahedral DNA‐RNA nanostructures (TDRN) in which P‐gp siRNA was precisely embedded in one edge of the TDRN. As shown in **Figure** **1**A, oligonucleotides (S1–S4) for TDRN assembly were mixed together in an equimolar ratio and the P‐gp siRNA‐organized TDRN was assembled step‐by‐steply and examined by 1.5% agarose gel electrophoresis. The one‐pot assembled TDRN was also characterized by dynamic light scattering (DLS) and transmission electron microscopy (TEM). The hydrodynamic diameter of the TDRN was measured to be 18.02 ± 0.12 nm (Figure 1B), and the average diameter of the TDRN in the dried state was 12.79 ± 0.21 nm, verified by TEM (Figure S2, Supporting Information).

After constructing P‐gp siRNA‐organized TDRN nanocarriers, we further optimized the loading conditions to encapsulate the chemotherapeutic drug Dox to generate a synergistic RNAi/Chemo‐therapy. Dox (3 mm) was incubated with the TDRN (6 µm) at 37 °C for 3 h, and the encapsulation efficiency (EE%) of TDRN@Dox was calculated to be 37%. The hydrodynamic diameter of the TDRN@Dox was measured to be 24.85 ± 0.35 nm (Figure 1B), and the average diameter of the TDRN@Dox in the dried state was 21.12 ± 0.18 nm, verified by TEM (Figure S2, Supporting Information). In order to improve the delivery efficiency of TDRN, we subsequently constructed TDRN@Dox@AuNC_p_ using PEI‐modified gold nanoclusters (AuNC@PEI) via electrostatic interaction. The positively charged AuNC@PEI with a size smaller than 3 nm was synthesized and characterized by TEM and fluorescence spectrum (Figure S3, Supporting Information). We applied DLS, TEM, and atomic force microscope (AFM) to analyze the formation of monodisperse nanostructures of TDRN@Dox@AuNC_p_. The hydrodynamic diameter of the TDRN@Dox@AuNC_p_ was measured to be 59.41 ± 0.30 nm (Figure 1B). The average diameter of TDRN@Dox@AuNC_p_ with a spherical shape verified by TEM and AFM was 55.86 ± 0.20 and 55.68 ± 0.73 nm, respectively (Figure 1D; Figure S2, Supporting Information). Zeta potential measurements were performed to characterize the TDRN (−7.8 mV), TDRN@Dox (−2.7 mV), and TDRN@Dox@AuNC_p_ (+8.3 mV, Figure 1C).

### 2.2 Analysis of RNase A and Mouse Serum Stability and Stimuli‐Responsive Release Capability of TDRN

**Figure 2:**
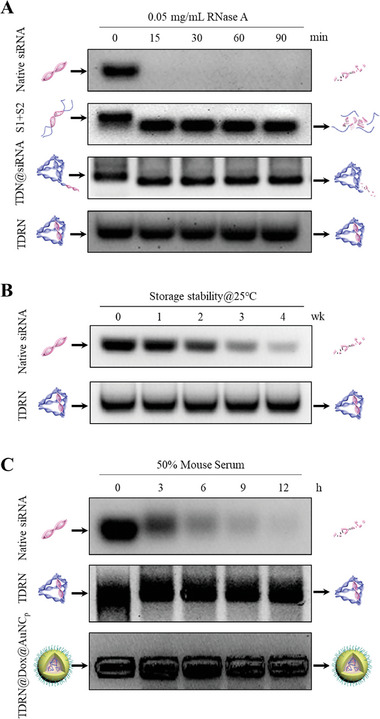
RNase A and long‐term storage stability and mouse serum stability of native siRNA and TDRN. A) The native siRNA, S1+S2, TDN@siRNA, and TDRN were incubated with 0.05 mg mL^−1^ RNase A for predetermined time intervals and analyzed on 1.5% agarose gels. B) The native siRNA and TDRN were mixed with PBS buffer and incubated for 0, 1, 2, 3, and 4 weeks and analyzed on 1.5% agarose gels. C) The native siRNA, TDRN, and TDRN@Dox@AuNC_p_ were incubated with 50% mouse serum for different time points, and determined by 1.5% agarose gels.

The RNase A stability of native siRNA, the duplex DNA‐SS‐RNA‐SS‐DNA strands (S1+S2), tail‐like TDN carrying siRNA via a sticky end (TDN@siRNA), and siRNA‐organized TDRN was evaluated without the use of cationic condensing species. The native siRNA, S1+S2, TDN@siRNA, and TDRN were incubated with 0.05 mg/mL RNase A solution at 37 °C for 15, 30, 60, and 90 min, respectively. As shown in **Figure** **2**A, native siRNA was rapidly degraded after 15 min incubation, as evidenced by 1.5% agarose gels. Meanwhile, two‐dimensional (2D) S1+S2 and tail‐like TDN@siRNA showed distinct misalignment of gel bands after 15 min RNase A incubation due to quick siRNA degradation. However, the dramatic improvement in RNase A stability of TDRN was determined in comparison to the above. No obvious degradation of siRNA in TDRN was observed even for 90 min RNase A incubation. The results demonstrated that the siRNA‐organized TDRN showed a significant improvement in RNase A stability due to the stable and highly rigid three‐dimensional (3D) nanostructure. In addition, the native siRNA and TDRN were incubated with PBS buffer and stored at room temperature (25 °C) for up to 4 weeks to investigate the long‐term storage stability. In contrast to the gradual degradation observed with native siRNA, TDRN exhibited excellent long‐term storage stability (Figure 2B). This study may provide new insights into the storage and transport of nucleic acid drugs, facilitating their application in challenging environments. To determine the ability of the TDRN and gold nanoclusters to protect siRNA from degradation by mouse serum nuclease, naked siRNA, TDRN, and TDRN@Dox@AuNC_p_ were incubated in 50% mouse serum at 37 °C for different time points (0, 3, 6, 9, and 12 h). Naked siRNA was quickly degraded in mouse serum, but the intensity of TDRN and TDRN@Dox@AuNC_p_ remained constant over 12 h incubation, indicating that the highly rigid nanostructure of TDRN and positively charged gold nanoclusters effectively protects siRNA from serum nuclease degradation (Figure 2C). Furthermore, the particle size of TDRN@Dox@AuNC_p_ remained almost constant during storage in PBS buffer for 3 days. And the particle size slightly increased to ≈80 nm in the 10% FBS solution due to the mild absorption of serum proteins from FBS, but the size uniformity was maintained within 3 days, indicating good stability in physiological environments (Figure S4, Supporting Information).

**Figure 3:**
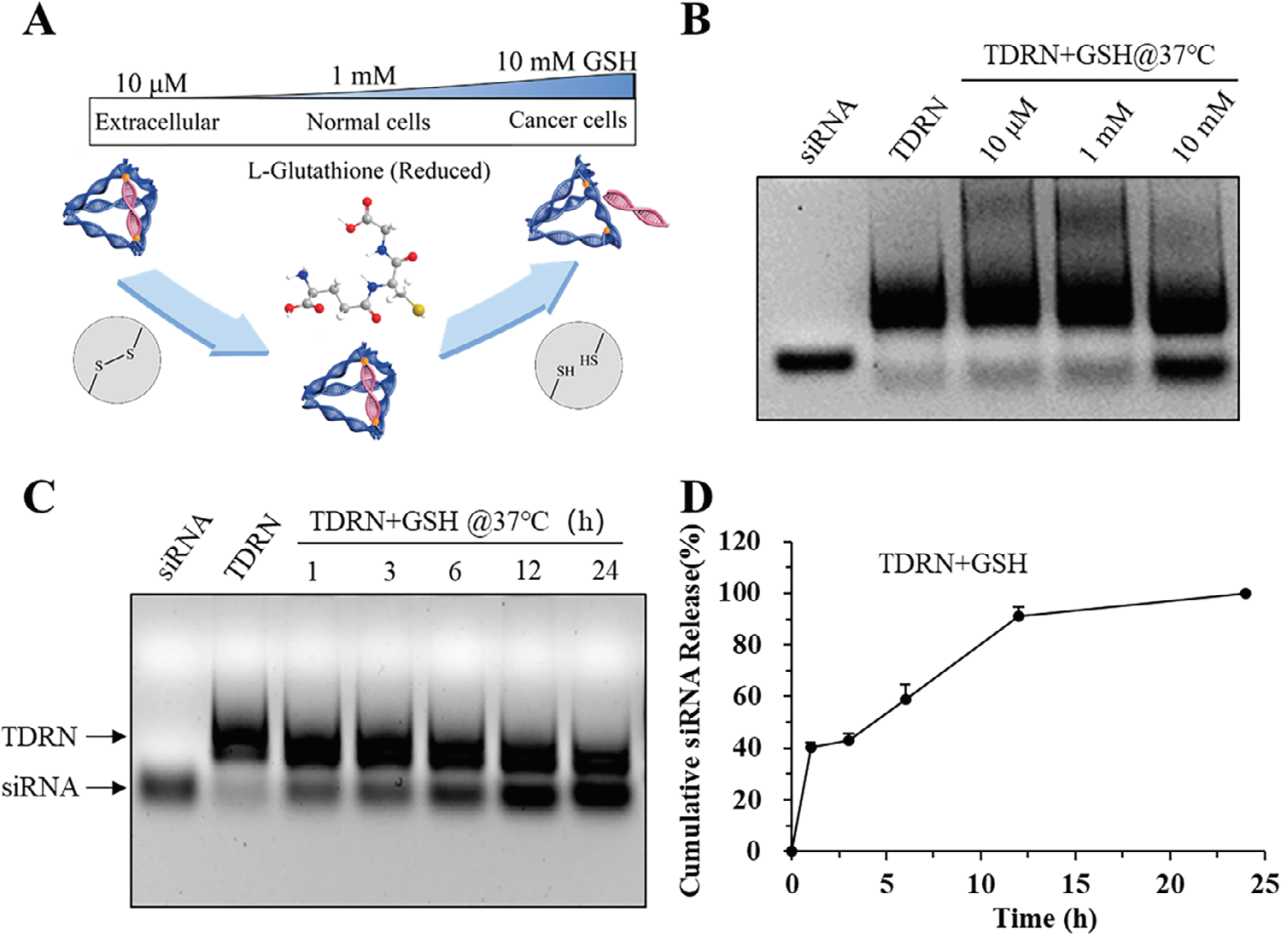
Analysis of the GSH‐responsive release properties of TDRN in solution. A) Schematic illustration of TDRN incubated with different GSH concentrations to simulate the exterior (10 µm) and interior (10 mm) of tumor cells as well as normal cells (1 mm). B) The TDRN was incubated with different GSH concentrations at 37 °C for 24 h and analyzed on 1.5% agarose gels. C) The TDRN was incubated with 10 mm GSH solution at 37 °C for predetermined time intervals. D) Percentages of cumulative siRNA release from the TDRN with 10 mM GSH solution were calculated using Image J software (n = 3).

The GSH‐responsive release properties of TDRN in solution were analyzed. The siRNA‐organized TDRN was incubated with different GSH concentrations (10 µm and 10 and 1 mm) to simulate the exterior and interior of tumor cells as well as normal cells, respectively. As expected, almost no siRNA was released from the TDRN in 10 µm and 1 mm GSH solution (**Figure** **3**B). As demonstrated in Figure 3C, the naked siRNA was released from the TDRN in the presence of 10 mm GSH, which moved to the same position in the gel as shown for the corresponding control siRNA. By extending the incubation time from 1 to 24 h, the high concentration of 10 mm GSH gradually triggered the release of naked siRNA from the TDRN until the release was complete (Figure 3C). The stimuli‐responsive release properties of TDRN were also analyzed in the dithiothreitol (DTT) solution. The cumulative siRNA release process of DTT was similar to that of GSH within the 24 h incubation (Figure S5, Supporting Information).

### 2.3 Cellular Uptake and Lysosomal Escape of TDRN@AuNC_p_

**Figure 4:**
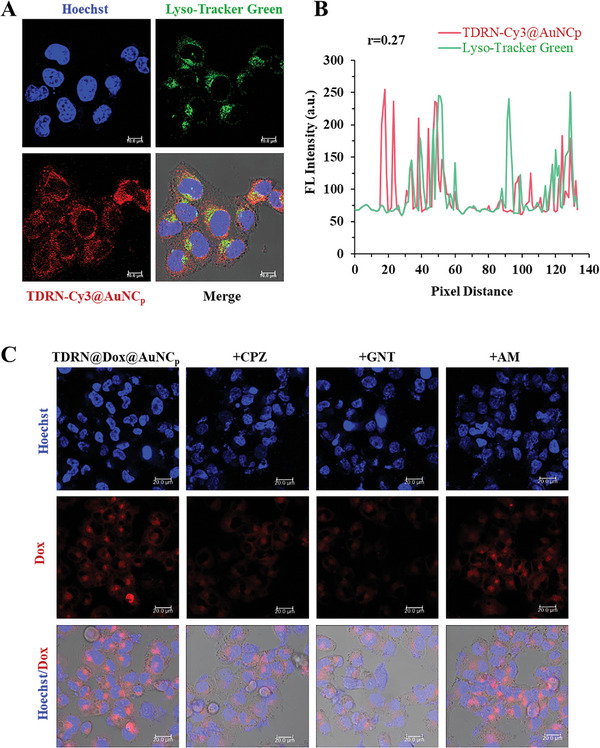
Analysis of lysosomal escape of Cy3‐labelled TDRN@AuNC_p_ and cellular uptake mechanism of TDRN@Dox@AuNC_p_ in HeLa/ADR cells using confocal laser scanning microscopy. A) Confocal microscopy images of TDRN‐Cy3@AuNC_p_ in HeLa/ADR cells incubated for 12 h. B) The Pearson's correlation coefficients between the green signals of Lyso‐Tracker and the red signals of TDRN‐Cy3@AuNC_p_. Scale bars = 10 µm. Hoechst (blue), Lyso‐Tracker (green), TDRN‐Cy3@AuNC_p_ (red). C). Confocal microscopy images of TDRN@Dox@AuNC_p_ pre‐treated with chlorpromazine (CPZ), amiloride (AM), genistein (GNT) for 1 h, and then transfected with TDRN@Dox@AuNC_p_ for 6 h. Scale bars = 20 µm. Hoechst (blue) and TDRN@Dox@AuNC_p_ (red).

To determine the cellular uptake efficiency of the TDRN@AuNC_p_, we used Cy3‐labelled TDRN@AuNC_p_ for flow cytometry analysis. As shown in Figure S6 (Supporting Information), the relative mean fluorescence intensity of HeLa/ADR cells treated with TDRN@AuNC_p_ with a mass ratio of 1 to 5 (TDRN: AuNC_p_) was highly stronger than of the TDRN‐treated cells, indicating that gold nanoclusters‐assisted delivery increased the cellular uptake of the TDRN. TDRN hardly entered the HeLa/ADR cells due to negative charges and lack of target‐ligand modifications. The TDRN@AuNC_p_ complex efficiently enhanced TDRN cellular internalization compared to free TDRN. We examined the lysosomal escape behavior of the Cy3‐labelled TDRN@AuNC_p_ in HeLa/ADR cells by fluorescence colocalization of the labeled TDRN and Lyso‐Tracker green using a confocal laser scanning microscope. Confocal microscopy images demonstrated that the Cy3 fluorescence intensity of TDRN‐Cy3@AuNC_p_ (150 nM siRNA equivalent) increased when the incubation time was prolonged from 6 to 12 h. The lysosome escape rate was investigated by Pearson colocalization analysis. As shown in **Figures** **4** and S7 (Supporting Information), the Pearson's correlation coefficients between the green signals from Lyso‐Tracker and the red signals from the labeled TDRN were all below 0.5 after treatment with TDRN‐Cy3@AuNC_p_ for 6 h or 12 h, revealing that the higher lysosome escape ability was due to the proton sponge effect of AuNC@PEI. Furthermore, the staggered linear distribution between the green and red signals verified that the TDRN‐Cy3@AuNC_p_ was located in the cytoplasm in HeLa/ADR cells. The cellular uptake pathway of TDRN@Dox@AuNC_p_ was then investigated using confocal microscopy. Briefly, treatment of HeLa/ADR cells with endocytosis inhibitors (chlorpromazine, amiloride, and genistein) was used to assess the effect on cellular uptake of TDRN@Dox@AuNC_p_ (Figure 4C). The lowest level of intracellular Dox red fluorescence was observed in the chlorpromazine and genistein treatments compared to the other groups, indicating that the cellular internalization pathway of TDRN@Dox@AuNC_p_ was mainly clathrin‐ and caveolin‐dependent endocytosis.

### 2.4 Evaluation of Gene Silencing Efficiency and Tumor Cell Killing Property of TDRN@Dox@AuNC_p_ in HeLa/ADR cells

**Figure 5:**
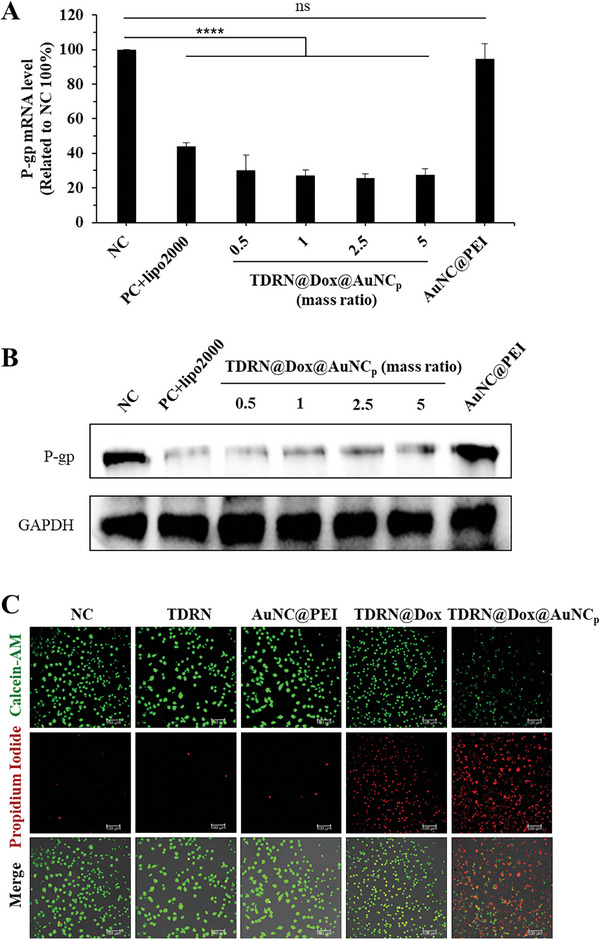
Gene silencing efficiency of TDRN@Dox@AuNC_p_ in HeLa/ADR cells. A) Real‐time qPCR analysis of target mRNA silencing efficiency with the TDRN@Dox@AuNC_p_ treatment for 48 h. B) Western blot analysis of protein expression levels of P‐gp and GAPDH with the TDRN@Dox@AuNC_p_ treatment for 48 h. C) Imaging of live and dead cells to verify the cytotoxicity of TDRN, AuNC@PEI, TDRN@Dox, and TDRN@Dox@AuNC_p_. Scale bars = 100 µm. Calcein‐AM (green), PI (red).

We further investigated the gene silencing efficiency of TDRN@Dox@AuNC_p_ in HeLa/ADR cells by real‐time qPCR and Western blot. As shown in **Figures** **5**A and S8 (Supporting Information), there was no influence on the relative P‐gp mRNA levels after treatment with AuNC@PEI. However, relative P‐gp mRNA levels were significantly decreased by treatment with TDRN@Dox@AuNC_p_ (150 nm siRNA equivalent) with the mass ratio of TDRN to AuNC@PEI varying from 0.5 to 5. Under the same experimental conditions, the efficiency of gene silencing in P‐gp protein expression levels was also determined using Western blot analysis. P‐gp protein expression levels in cells treated with TDRN@Dox@AuNC_p_ were similar to those of the positive control group using the Lipofectamine 2000 transfection reagent with a successful decrease at 150 nm (Figure 5B). The gene silencing efficiency of TDRN@AuNC_p_ was similar to that of TDRN@Dox@AuNC_p_, indicating that the effect of Dox on gene silencing can be excluded (Figure S9, Supporting Information). The TDN@Dox@AuNC_p_ control group, without the siRNA embedded in the TDN skeleton, showed no gene silencing. All these results demonstrated that TDRN@Dox@AuNC_p_ was successfully used to achieve gene silencing efficiency of P‐gp gene expression in GSH‐responsive HeLa/ADR cells. However, the TDRN@AuNC_p_ and TDRN@Dox@AuNC_p_ groups had no gene silencing efficiency in normal human umbilical vein endothelial cells (HUVEC) treated with 0.16 µg mL^−1^ Dox for 14 days to promote P‐gp protein expression (Figure S9, Supporting Information). This result indicates that the low concentration of GSH in normal HUVEC cells is not sufficient to respond to the release of siRNA from the TDRN skeleton, which is consistent with the results of our GSH‐responsive release properties in solution.

Furthermore, live and dead cell imaging was performed by confocal imaging analysis using the co‐staining agents calcein‐AM and propidium iodide (PI) (Figure 5C). The highest level of dead cells as shown by red fluorescence was observed in the TDRN@Dox@AuNC_p_ treatment (150 nm siRNA, 5.6 µm Dox equivalent) compared to the other groups, suggesting that the combination of RNAi therapy and chemotherapy was more effective.

### 2.5 Synergistic RNAi/Chemo‐Therapy of TDRN@Dox@AuNC_p_ in MDR Tumor Cells

**Figure 6:**
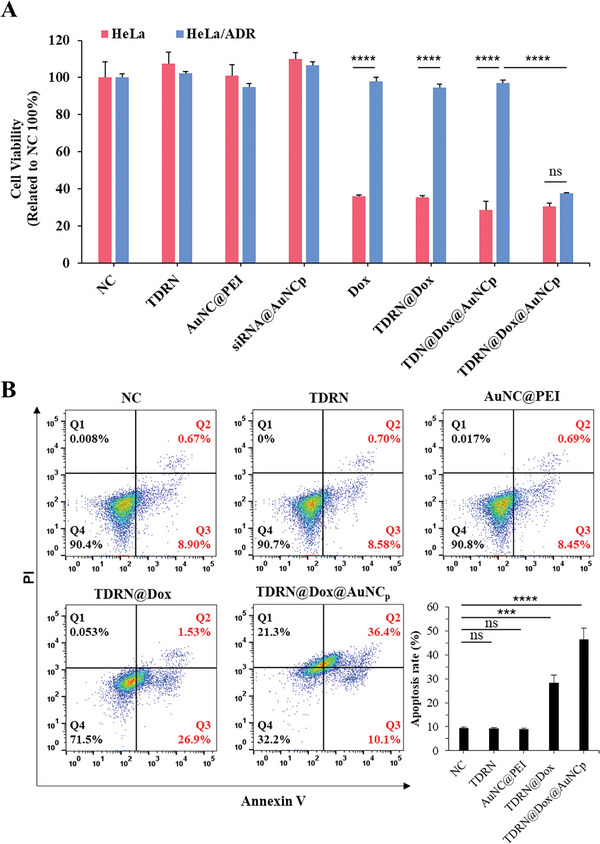
Synergistic RNAi/Chemo‐therapy of TDRN@Dox@AuNC_p_ in HeLa/ADR cells. A) Cell viability of HeLa and HeLa/ADR cells treated with TDRN, AuNC@PEI, siRNA@AuNC_p_, Dox, TDRN@Dox, TDN@Dox@AuNC_p_ and TDRN@Dox@AuNC_p_, respectively. B) Cell apoptosis analysis in HeLa/ADR cells treated with TDRN@Dox@AuNC_p_ determined by flow cytometry using Annexin V‐FITC/PI double staining assay and the apoptosis rates of HeLa/ADR cells were calculated using FlowJo software.

We subsequently investigated the ability of TDRN@Dox@AuNC_p_ to inhibit the proliferation of HeLa/ADR cells, as confirmed by the MTT assay. As shown in **Figures** **6**A and S10 (Supporting Information), neither the biocompatible TDRN, nor the AuNC@PEI, nor the siRNA@AuNC_p_ alone showed any observable cytotoxicity at the indicated treatment. Treatment of Dox‐sensitive HeLa cells with 5.6 µm Dox, 150 nm TDRN@Dox (corresponding to Dox 5.6 µm), and the TDN@Dox@AuNC_p_ control group, without the siRNA embedded in the TDN skeleton, for 24 h resulted in more than 60% cytotoxicity. In contrast, more than 95% of drug‐resistant HeLa/ADR cells survived under the same treatment. However, we observed a significant improvement in the proliferation inhibition efficiency of TDRN@Dox@AuNC_p_ in HeLa/ADR cells with almost 60% cytotoxicity. These results demonstrate that TDRN@Dox@AuNC_p_, an RNAi therapy targeting P‐gp in combination with chemotherapy, overcomes multiple drug resistance in HeLa/ADR cells in vitro. We further evaluated the apoptotic cells using Annexin V‐FITC/PI double staining assay by flow cytometry analysis to investigate whether TDRN@Dox@AuNC_p_ inhibited the proliferation of drug‐resistant HeLa/ADR cells and drug‐sensitive HeLa cells through the mechanism of apoptosis (Figure 6B; Figure S11, Supporting Information). The HeLa/ADR cell results demonstrated that the apoptosis rates induced by TDRN@Dox@AuNC_p_ (corresponding to Dox 5.6 µM) were 46.5%. TDRN@Dox@AuNC_p_ had the highest apoptosis rate of HeLa/ADR cells among all groups at the same indicated dosages. The evaluation of apoptotic cells of TDRN@Dox@AuNC_p_ with normal drug‐sensitive HeLa cells was performed as a control in the flow cytometry experiments (Figure S11, Supporting Information).

### 2.6 In Vivo Biodistribution of TDRN@Dox@AuNC_p_

**Figure 7:**
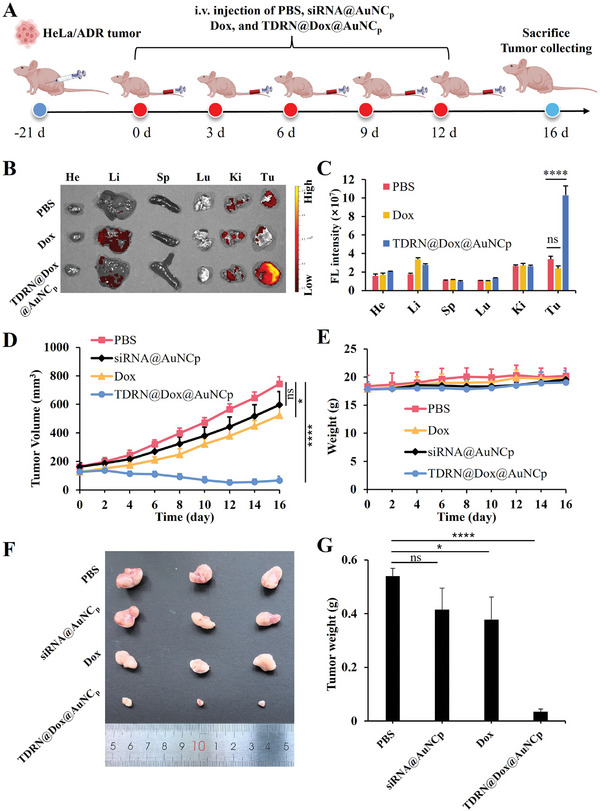
In vivo biodistribution and antitumor effects of TDRN@Dox@AuNC_p_. A) Schematic illustration of the treatment schedules. B) In vivo biodistribution of TDRN@Dox@AuNC_p_ in HeLa/ADR tumor‐bearing nude mice. Fluorescence imaging of tumor tissues (Tu) and major organs (He: heart; Li: liver; Sp: spleen; Lu: lung; and Ki: kidney) harvested 12 h after tail vein injection. C) Quantitative analysis of Dox fluorescence in tumor and major tissues. D) Tumor volumes of HeLa/ADR tumor‐bearing mice during 16 days of observation. E) Body weight of HeLa/ADR tumor‐bearing mice. F) Photograph of excised tumor tissues at day 16. G) Weight of excised tumor tissues.

Motivated by the good in vitro properties of TDRN@Dox@AuNC_p_, we then evaluated its in vivo performance. We established a HeLa/ADR tumor xenograft model in BALB/c nude mice to examine the in vivo biodistribution of TDRN@Dox@AuNC_p_ using animal imaging systems to observe the Dox fluorescence signal. After 12 h of i.v. administration, mice were sacrificed for ex vivo fluorescence imaging of major organs and tumor tissues. As shown in **Figure** **7**B, TDRN@Dox@AuNC_p_ showed a stronger Dox signal in the tumor region compared to the PBS and free Dox groups. The signal of Dox was mainly observed in the tumor tissues after TDRN@Dox@AuNC_p_ treatment due to the EPR effect favored by their particle size. This was also confirmed by quantitative analysis of fluorescence intensity (Figure 7C). The results demonstrated the sustained metabolism and excellent tumor‐target accumulation of TDRN@Dox@AuNC_p_ compared to free Dox treatment, providing the foundation for subsequent synergistic RNAi/Chemo‐therapy in vivo.

### 2.7 Synergistic RNAi/Chemo‐Therapy of TDRN@Dox@AuNC_p_ In Vivo

We evaluated the synergistic effects of RNAi/Chemo‐therapy against multidrug‐resistant tumors (HeLa/ADR) in vivo. HeLa/ADR cells were injected subcutaneously 21 days prior to treatment. From day 0, HeLa/ADR tumor‐bearing mice were administered with PBS, siRNA@AuNC_p_, free Dox, and TDRN@Dox@AuNC_p_ through tail vein injection at equivalent doses of 1 nmol siRNA and/or 2 mg kg^−1^ Dox every 3 days for 5 consecutive treatments (Figure 7A). As shown in Figure 7D, tumor volumes of the PBS, siRNA@AuNC_p_, and free Dox groups reached 745, 597, and 522 mm^3^ at day 16, respectively. A slight tumor growth inhibitory effect was observed with siRNA@AuNC_p_ and free Dox groups, which can be attributed to the limitations of only P‐gp gene silencing and only Dox administration in the resistance of the HeLa/ADR tumor model to Dox, respectively. While the TDRN@Dox@AuNC_p_ treated group showed the most effective tumor suppression (67 mm^3^) with the percentage of tumor growth inhibition (% inh) at 91.0% and tumor growth shrinkage (% reg) at 47.4%. In addition, no significance was observed in the weight of the mice in each group (Figure 7E), demonstrating the biocompatibility of AuNC@PEI and TDRN@Dox@AuNC_p_ in vivo. No apparent pathological changes were observed in histological sections of major organs (heart, liver, spleen, lung, and kidney) during the TDRN@Dox@AuNC_p_ treatment, indicating no obvious systemic toxicity from the TDRN@Dox@AuNC_p_ treatment (Figure S12, Supporting Information).

**Figure 8:**
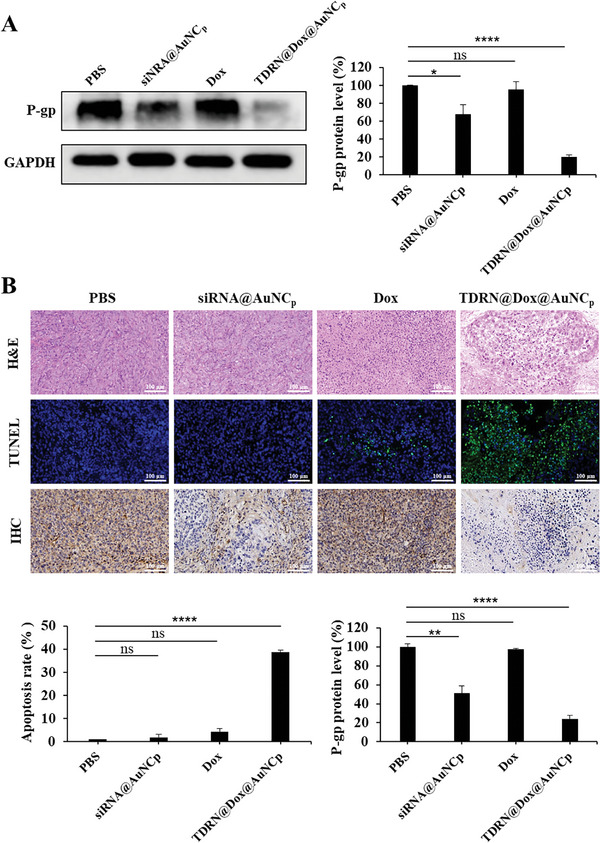
Evaluation of the therapeutic effect of TDRN@Dox@AuNC_p_. A) Western blot analysis of P‐gp protein expression in excised tumor tissues after treatment with PBS, siRNA@AuNC_p_, free Dox, and TDRN@Dox@AuNC_p_. B) H&E, TUNEL (green fluorescence), and IHC (P‐gp‐positive cells were stained brown) analysis of HeLa/ADR xenograft tumors after the indicated administration. Scale bars = 100 µm.

To subsequently evaluate the therapeutic effect of TDRN@Dox@AuNC_p_, we analyzed the excised tumor tissues. Four days after administration, both Western blot and immunohistochemistry (IHC) results demonstrated significant P‐gp protein silencing in the siRNA@AuNC_p_ and TDRN@Dox@AuNC_p_ groups, whereas the free Dox group showed no apparent inhibition of P‐gp protein levels (**Figure** **8**A,B). These results confirm the more effective suppression of P‐gp drug efflux pumps by TDRN@Dox@AuNC_p_ in vivo than that of the siRNA@AuNC_p_, probably due to the higher stability and improved long‐term gene silencing properties of tetrahedral DNA‐RNA nanocages. Hematoxylin and eosin (H&E) staining demonstrated massive cytoplasmic vacuolization and nuclear disintegration in the TDRN@Dox@AuNC_p_ group. TdT‐mediated dUTP nick end labeling (TUNEL) staining also showed the highest tumor cell necrosis as well as late apoptosis in the TDRN@Dox@AuNC_p_ group compared to the others, which was consistent with the H&E assay (Figure 8B). Taken together, these data demonstrate that the biocompatible TDRN@Dox@AuNC_p_ effectively inhibits tumor growth through synergistic RNAi/Chemo‐therapy of MDR tumors.

## 3 Conclusion

In summary, we have designed and constructed stimuli‐responsive programmable tetrahedral DNA‐RNA nanocages embedded with RNAi and chemo‐drugs for synergistic RNAi/Chemo‐therapy. These self‐assembled TDRN nanocarriers with gold nanocluster‐assisted delivery showed several advantages for drug co‐delivery. First, the structurally well‐defined TDRN, with significantly improved RNase A, long‐term storage, and mouse serum stability, is tailored to the precise organization of the stimuli‐responsive P‐gp siRNA element in one edge of the DNA tetrahedron. Second, the Dox‐loaded TDRN nanocages TDRN@Dox@AuNC_p_ with high encapsulation efficiency can achieve efficient cellular internalization and higher lysosomal escape in cells. Finally, the multifunctional TDRN@Dox@AuNC_p_ possesses effective proliferation inhibition efficiency via the mechanism of apoptosis to overcome multiple drug resistance in HeLa/ADR cells. In vivo experiments also demonstrate that TDRN@Dox@AuNC_p_ can selectively and effectively co‐deliver RNAi/Chemo‐drugs into multidrug resistance tumor with a synergistic antitumor effect. These results indicate that biocompatible and programmable tetrahedral DNA‐RNA nanocages woven with stimuli‐responsive siRNA hold great promise as a novel combination approach for synergistic RNAi/Chemo‐therapy of MDR tumor in vitro and in vivo. The construction of two‐in‐one or multi‐in‐one stimuli‐responsive TDRNs may be a promising strategy to explore the simultaneous silencing of multiple resistance proteins to synergistically reverse tumor multidrug‐resistance.

## 4 Experimental Section

### Synthesis and Purification of DNA‐SS‐RNA‐SS‐DNA Oligonucleotides with Two Disulfide Bond Modifications

The oligonucleotides used in this study for TDRN assembly are listed in Table S1 (Supporting Information). Oligonucleotides were synthesized according to standard phosphoramidite chemistry using a LK‐48E DNA/RNA solid‐phase synthesizer. DNA‐SS‐RNA‐SS‐DNA oligonucleotides were synthesized by sequentially coupling with 38‐nt DNA nucleotides, the phosphoramidites of the thiol‐modified C6 S‐S, 19‐nt RNA nucleotides, the phosphoramidites of the thiol‐modified C6 S‐S and 15‐nt DNA nucleotides, respectively. These synthetic oligonucleotides were cleaved from solid CPGs by ammonium hydroxide/methylamine solution, followed by removal of TBDMS by the Et3N·3HF. Oligonucleotides were precipitated and followed collected by centrifugation. These synthetic oligonucleotides were further purified by HPLC and characterized using ESI‐MS (see the Supporting Information).

### Assembly of TDRN

Four oligonucleotides (S1–S4) for TDRN assembly were mixed in 1×TAE/Mg^2+^ buffer (40 mm Tri, 40 mm acetic acid, 50 mm EDTA, and 12.5 mm MgCl_2_·6H_2_O) in equimolar quantities, incubated at 95 °C for 5 min for denaturation, and subsequently cooled from 95 °C to 4 °C within 30 s and kept at 4 °C for 2 h for complementary pairing.

### Preparation of Dox‐Loaded TDRN

For Dox intercalation into the TDRN, Dox (3 mm) was mixed with the TDRN (6 µm) at 37 °C and stirred at 400 rpm for 3 h. After the loading process, the TDRN@Dox red solution was then purified through dialysis against DEPC‐treated water (Mw = 5000 kDa cut‐off). The content of intercalated Dox molecules per TDRN was measured by the absorption at a wavelength of 480 nm using a UV–vis spectrophotometer. The encapsulation efficiency (EE%) of TDRN to Dox was calculated using the equation: EE% = [(Total amount of Dox‐free Dox)/Total amount of Dox] × 100%.

### Preparation of TDRN@Dox@AuNC_p_

The fluorescent and low‐molecular‐weight PEI‐modified gold nanoclusters (AuNC_p_) had been synthesized. In brief, PEI‐lipoic acid conjugates were synthesized by coupling lipoic acid to 1.8 kDa PEI via carbodiimide chemistry. Subsequently, a molar ratio of PEI‐lipoic acid conjugates to HAuCl_4_·3H_2_O solution (100 mm) of 6:1 and aqueous NaOH solution (2 m) were mixed with ultrapure water. The mixed solution was heated to 95 °C with stirring for 24 h. An aqueous solution of AuNC@PEI with yellow color was formed and further purified by dialysis. Finally, the positively charged AuNC@PEI (1 mg mL^−1^) was mixed with TDRN@Dox solution as prepared above to form TDRN@Dox‐AuNC@PEI complex via electrostatic interaction. The prepared complex was abbreviated as TDRN@Dox@AuNC_p_.

### Characterization of TDRN, TDRN@Dox and TDRN@Dox@AuNC_p_

Zeta potential, particle size, and polydispersity index of the TDRN, TDRN@Dox, and TDRN@Dox@AuNC_p_ were determined using DLS on a Zetasizer (NanoZS, Malvern). TEM images of the TDRN, TDRN@Dox, and TDRN@Dox@AuNC_p_ were obtained using a Tungsten filament transmission electron microscope (JEM‐1200EX, JEOL). AFM was also used to characterize TDRN@Dox@AuNC_p_ (Dimension ICON, Bruker).

### Agarose Gel Analysis

The assembly of TDRN, RNase A, and mouse serum stability assay as well as stimuli‐responsive release efficiency of TDRN were examined using 1.5% agarose gels in the study. The agarose gels were pre‐stained with Gel‐red stain and run for 15 min at 200 V in RNase‐free 1×TAE buffer. Gel images were captured using gel imaging systems (ChemiDoc, Bio‐Rad).

### RNase A Stability, Storage Stability, and Mouse Serum Stability Assay

The TDN@siRNA complex had the siRNA located at the vertex outside of the nanocage, with one extended DNA end forming a complementary base pairing with the TDN. The equimolar native siRNA, S1+S2, TDN@siRNA, and TDRN were labeled with Cy3 and then incubated with 0.05 mg/mL RNase A at 37 °C in an incubator for 0, 15, 30, 60, and 90 min, respectively. Each prepared sample was determined using agarose gel analysis. For storage stability assay, the equimolar native siRNA and TDRN were mixed with PBS buffer and incubated at 25°C for 0, 1, 2, 3, and 4 weeks, respectively. To assess mouse serum stability, native siRNA, TDRN, and TDRN@Dox@AuNC_p_ were incubated with 50% mouse serum at 37 °C for 0, 3, 6, 9, and 12 h. To determine the stability of TDRN@Dox@AuNC_p_ in a complex physiological environment, DLS was used to measure the average particle size at 0, 12, 24, 48, and 72 h after storage in PBS buffer and 10% FBS solution at 37 °C, respectively.

### Stimuli‐Responsive Release Efficiency of TDRN

TDRN was incubated with the DTT solution (5 mm) or GSH solution (reduced L‐glutathione, 10 mm) in 1×TAE/Mg^2+^ buffer for 0, 1, 3, 6, 12, and 24 h at 37 °C, respectively. TDRN was incubated with GSH at 37°C in an incubator for 24 h with GSH concentrations of 10 µm and 1 and 10 mm, respectively. Each prepared sample was determined using agarose gel analysis.

### Flow Cytometry Analysis

HeLa/ADR cells were seeded overnight in 12‐well plates and treated with the Cy3‐labeled siRNA/ Lipofectamine 2000 (150 nm siRNA) and the Cy3‐labeled TDRN@AuNC_p_ (150 nM siRNA equivalent), where the mass ratio of TDRN to AuNC@PEI varied from 1 to 5, for 6 h, respectively.

### Confocal Imaging Analysis

HeLa/ADR cells were plated on a confocal dish overnight, then treated with Cy3‐labelled TDRN@Dox@AuNC_p_ (150 nM siRNA equivalent) for 6 or 12 h, respectively. After incubation, HeLa/ADR cells were washed with PBS for three times. The cells were stained with 10 µg mL^−1^ Hoechst 33342 (E_x_ = 405 nm), and 50 nM Lyso Tracker Green (E_x_ = 488 nm) for 30 min at 37 °C. In the cellular uptake mechanism study, HeLa/ADR cells were pretreated with the endocytosis inhibitors consisting of chlorpromazine (CPZ, 0.2 mm), amiloride (AM, 20 mm), and genistein (GNT, 2 mm) for 1 h, respectively. After inhibitor pretreatment, the cells were transfected with the TDRN@Dox@AuNC_p_ (150 nM siRNA equivalent, mass ratio = 1:1) for 6 h. The cells were washed with PBS and stained with 10  µg mL^−1^ Hoechst 33342 (Ex = 405 nm) for 30 min at 37 °C. For investigating live and dead cell tests, HeLa/ADR cells were seeded overnight on glass‐bottomed culture dishes, and AuNC@PEI, TDRN@Dox, TDRN@Dox@AuNC_p_ were applied at the concentration of 150 nm siRNA (corresponding to Dox 5.6 µm). After 24 h incubation, the cells were incubated with fresh medium containing 2 mm calcein‐AM and 1.5 mm PI for 20 min at 37 °C. Cell imaging was investigated using a confocal laser scanning microscope (Leica SP5).

### RT‐PCR and Real‐Time Quantitative PCR

HeLa/ADR cells were transfected with positive siRNA (150 nM) using the Lipofectamine 2000 transfection reagent or TDRN@Dox@AuNC_p_ (150 nm siRNA equivalent), with the mass ratio of TDRN to AuNC@PEI varied from 0.5 to 5, respectively. After 6 h of incubation, cells were replaced with fresh advanced DMEM for another 48 h incubation. Total RNA was extracted from HeLa/ADR cells using the Trizol reagent (Vazyme Biotech Co., Ltd.). RNA was reverse transcribed into the cDNA using HiScript Q Select RT SuperMix reagent (+gDNA wiper). Obtained cDNA was used as a template for RT‐PCR or real‐time qPCR amplification according to the manufacturer's protocol. Real‐time qPCR was performed using SYBR Premix Ex TaqTM II (Yeasen Biotech). GAPDH was used as an internal control. Primer sequences of P‐gp and GAPDH are listed as follows:

P‐gp forward primer: 5ʹ‐GCTCATCGTTTGTCTACAGTTCG‐3ʹ and reverse primer: 5ʹ‐ATTTCCAAGGCATCAATTTCAC‐3ʹ;

GAPDH forward primer: 5ʹ‐GACTCATGACCACAGTCCATGC‐3ʹ and reverse primer: 5ʹ‐AGAGGCAGGGATGATGTTCTG‐3ʹ.

### Western Blotting

Total cellular proteins were extracted from TDRN@Dox@AuNC_p_ treated HeLa/ADR cells and HUVEC cells using RIPA lysis buffer and quantified using the BCA protein assay kit. For in vivo Western blot assay, tumor tissues were homogenized and total protein was also isolated using RIPA lysis buffer. Protein extracts (20 µg) for each treatment were separated on 8% SDS‐PAGE, and then transferred onto the PVDF membrane. The PVDF membrane was blocked using 5% milk for 0.5 h and then incubated with rabbit monoclonal antibody against P‐gp (Abcam, ab170904) and mouse monoclonal antibody against GAPDH (Beyotime, AF2819) at 4 °C overnight. After washing three times, the PVDF membrane was incubated with HRP‐labelled anti‐rabbit or mouse secondary antibody (Beyotime, A0208, and A2016) for 1 h, respectively. The membrane was probed using an ECL hypersensitive luminescent liquid and imaged by a ChemDoc imaging system.

### Measurement of Cell Viability

HeLa and HeLa/ADR cells were plated on 96‐well plates and cultured overnight. The cells were treated with PBS, TDRN, AuNC@PEI, siRNA@AuNC_p_, Dox, TDRN@Dox, TDN@Dox@AuNC_p_, and TDRN@Dox@AuNC_p_ at the concentration of 150 nM siRNA (corresponding to Dox 5.6 µm), respectively. After incubation for 24 h, the cells were replaced with the fresh advanced medium containing 0.5 mg mL^−1^ MTT and incubated for another 4 h at 37°C. Then, 100 µL DMSO in each well was resolved formazan crystals after replacement MTT solution. After shaking for 20 min, the absorbance was measured at 570 nm to calculate cell viability. Each experiment was performed in three replicates.

### Apoptosis of TDRN@Dox@AuNC_p_

HeLa and HeLa/ADR cells were seeded in 12‐well plates and cultured overnight. The cells then were treated with TDRN@Dox@AuNC_p_ at the concentration of 150 nm siRNA (corresponding to Dox 5.6 µm) for 8 h, respectively. The cells were replaced with the fresh advanced medium for another 24 h incubation. The apoptosis of HeLa and HeLa/ADR cells was measured using an apoptosis analysis kit containing annexin V and PI (Beyotime, C1062M) according to the manufacturer's instructions. The data was measured by a flow cytometer (FC500 Beckman, USA) and analyzed by FlowJo V10 software.

### Biodistribution of TDRN@Dox@AuNC_p_

HeLa/ADR tumor‐bearing mice were administered with equivalent doses of free Dox or TDRN@Dox@AuNC_p_ (corresponding to 1 nmol siRNA, 2 mg kg^−1^ Dox) via tail vein injection. Fluorescence imaging was captured through an animal imaging system at 12 h after injection under isoflurane gas anesthesia. The mice were sacrificed to collect the major organs and tumors for ex‐vivo fluorescence imaging (IVIS Spectrum, USA).

### HeLa/ADR Tumor‐Bearing Mice Model

Female BALB/c nude mice (15‐16 g, 5 weeks old) were purchased from Shanghai SLAC Laboratory Animal Co., Ltd (Shanghai, China). All animal experiments were approved by the Laboratory Animal Welfare & Ethics Committee of Fujian Medical University (IACUC FJMU 2023‐0209). Briefly, the HeLa/ADR tumor‐bearing mice model was established by injecting ≈1.0 × 10^7^ HeLa/ADR cells suspended in PBS solution and mixed with an equal volume of Matrigel (Beyotime, C0387) in the right flanks of BALB/c nude mice. When the tumor volume was ≈150 mm^3^, mice were randomly divided into 4 groups (n = 3 per group). The mice were administered with 0.9% PBS, free Dox, siRNA@AuNC_p_, and TDRN@Dox@AuNC_p_ at an equivalent dose (corresponding to 1 nmol siRNA, 2 mg kg^−1^ Dox) via tail vein injection in a 150 µL every 3 days for 5 treatments. The tumor size was measured every 2 days using a digital vernier caliper across the longest (a) and shortest (b) diameter. The tumor volume was calculated using the formula of V = 0.5·a·b^2^. The weight of the mice was measured using an electronic balance. After 16 days of treatment, the tumor‐bearing mice were sacrificed. The percentage of tumor growth inhibition (% inh) and tumor growth shrinkage (% reg) were calculated as follows: % inh = (TV_cont_‐TV_treat_)/TV_cont_×100; % reg = (TV_start_‐TV_treat_)/TV_start_×100. TV_cont_ is the mean tumor volume of the PBS control group, TV_start_ represents the mean tumor volume of the treated group at the initiation of treatment, and TV_treat_ is the mean tumor volume of the treated group at the end of treatment. The tumor tissues were imaged and weighted and then stained with TUNEL for apoptosis level and IHC for P‐gp protein levels. Western blot was performed to evaluate the P‐gp protein expression levels in tumor tissues. The tumors and the major organs (heart, liver, lung, spleen and kidney) were harvested and examined by H&E staining.

### Statistical Analysis

All the data were expressed as mean ± standard deviation (SD) obtained from at least three samples. Statistical significance was analyzed using a one‐way analysis of variance (for multiple‐group analysis) and a two‐tailed Student's *t*‐test (for two‐group comparison). A value of *p *< 0.05 was defined as statistically significant.

## Conflict of Interest

The authors declare no conflict of interest.

## Supporting information

Supporting Information

## Data Availability

The data that support the findings of this study are available from the corresponding author upon reasonable request.
