# Supplementary material for: Programmable Tetrahedral DNA‐RNA Nanocages Woven with Stimuli‐Responsive siRNA for Enhancing Therapeutic Efficacy of Multidrug‐Resistant Tumors
Source: Adv Sci (Weinh). 2024 Jun 25;11(32):2404112. doi: 10.1002/advs.202404112 (PMC11348235; doi:10.1002/advs.202404112)

**Supporting Information**

**Programmable Tetrahedral DNA-RNA Nanocages Woven with Stimuli-Responsive siRNA for Enhancing Therapeutic Efficacy of Multidrug-Resistant Tumors**

Changmai Chen^1,^^+^, Maocheng Yu^1,+^, Qing Li^1^, Ying Zhou^1^, Mengting Zhang^1^, Shanyu Cai^1^, Jiaojiao Yu^1^, Zhongnan Huang^1^, Jiaan Liu^1^, Ye Kuang^1^, Xinjing Tang^2^, and Wei Chen^1,^*

^1^ Fujian Key Laboratory of Drug Target Discovery and Structural and Functional Research, School of Pharmacy, Fujian Medical University, Fuzhou 350122, China

^2^ State Key Laboratory of Natural and Biomimetic Drugs, School of Pharmaceutical Sciences, Peking University, Beijing 100191, China

^+^ C.C. and M.Y. contributed equally

* Correspondence

Prof. Dr. Wei Chen,

Fujian Key Laboratory of Drug Target Discovery and Structural and Functional Research, School of Pharmacy, Fujian Medical University, Fuzhou 350122, China

Tel: +86-0591-22862016

Email: weichen@fjmu.edu.cn

**Table S1.** The sequences of oligonucleotides used in this study for TDRN assembly.

**
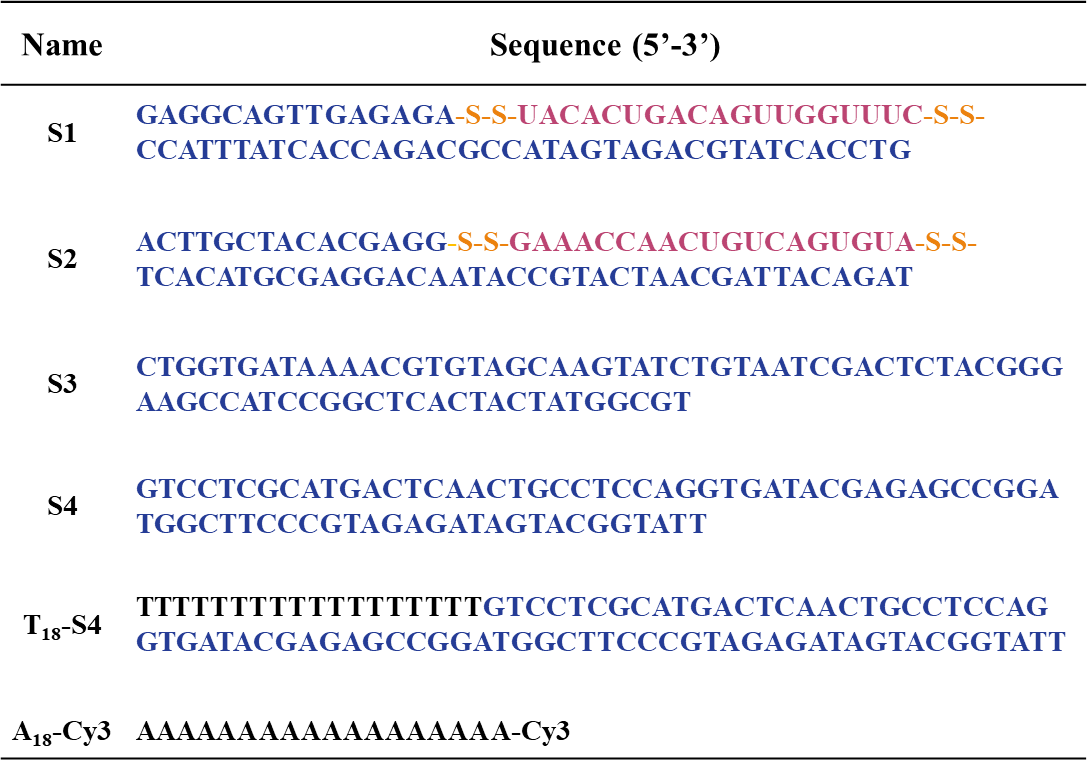
**

**
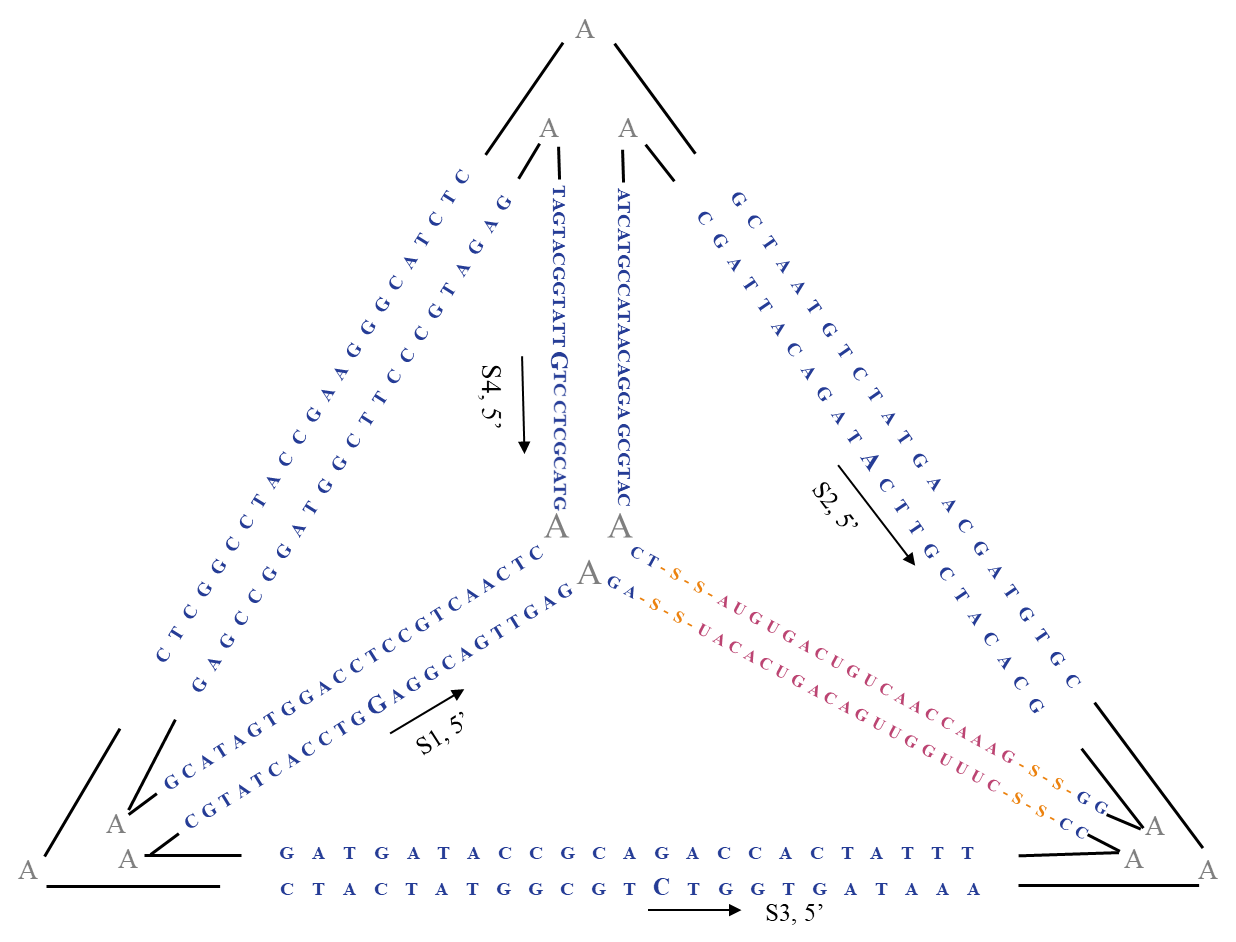
**

**Figure S1.** Illustration of structure and sequence of the Tetrahedral DNA-RNA Nanocages.

**
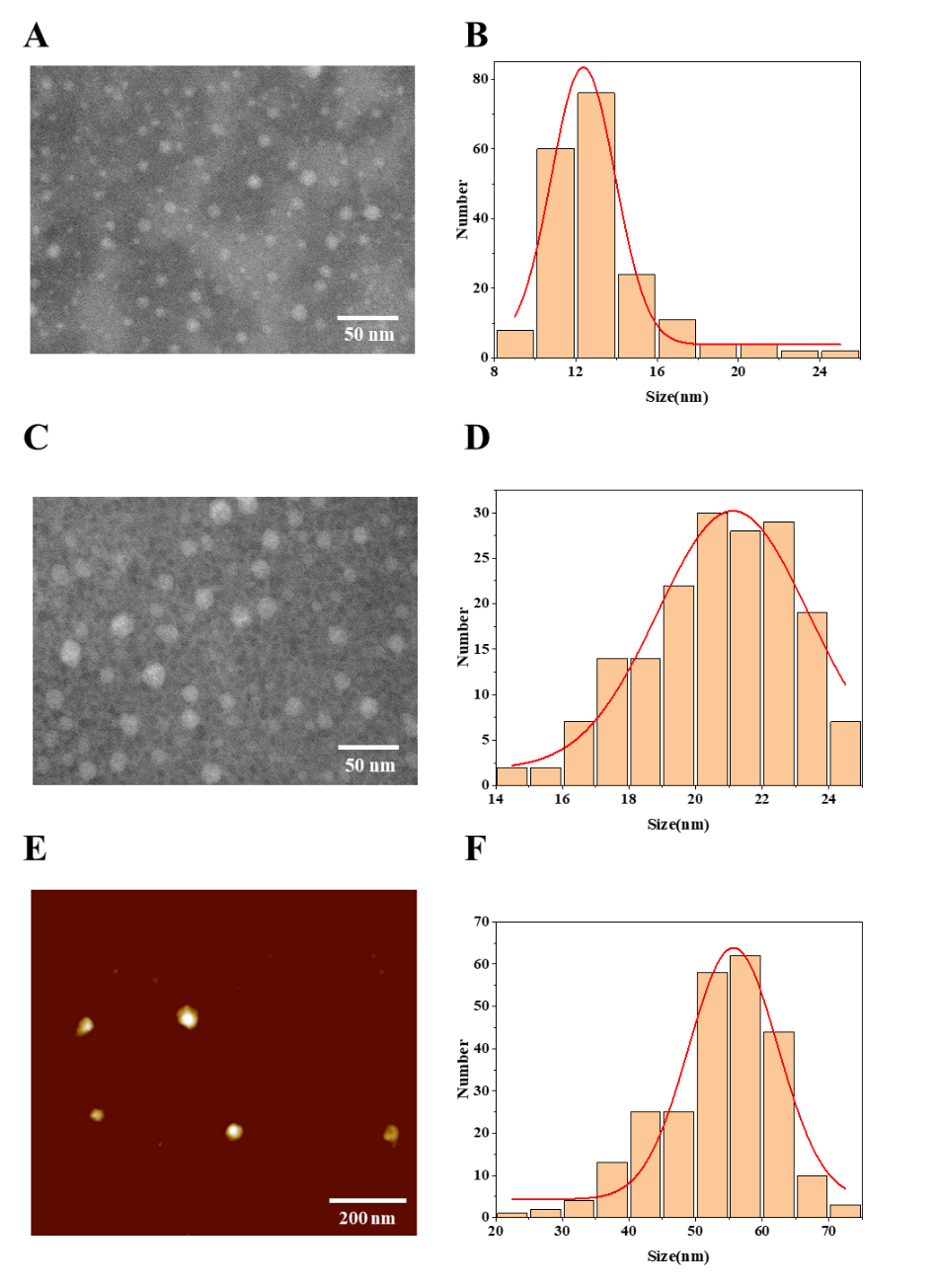
Figure S2.** Characterization of TDRN, TDRN@Dox and TDRN@Dox@AuNC_p_. A) TEM characterization of TDRN. B) Particle size distribution of TDRN in the dried calculated to TEM images. C) TEM characterization of TDRN@Dox. D) Particle size distribution of TDRN@Dox in the dried calculated to TEM images. E) AFM characterization of TDRN@Dox@AuNC_p_. F) Particle size distribution of TDRN@Dox@AuNC_p_ calculated to AFM images.

**
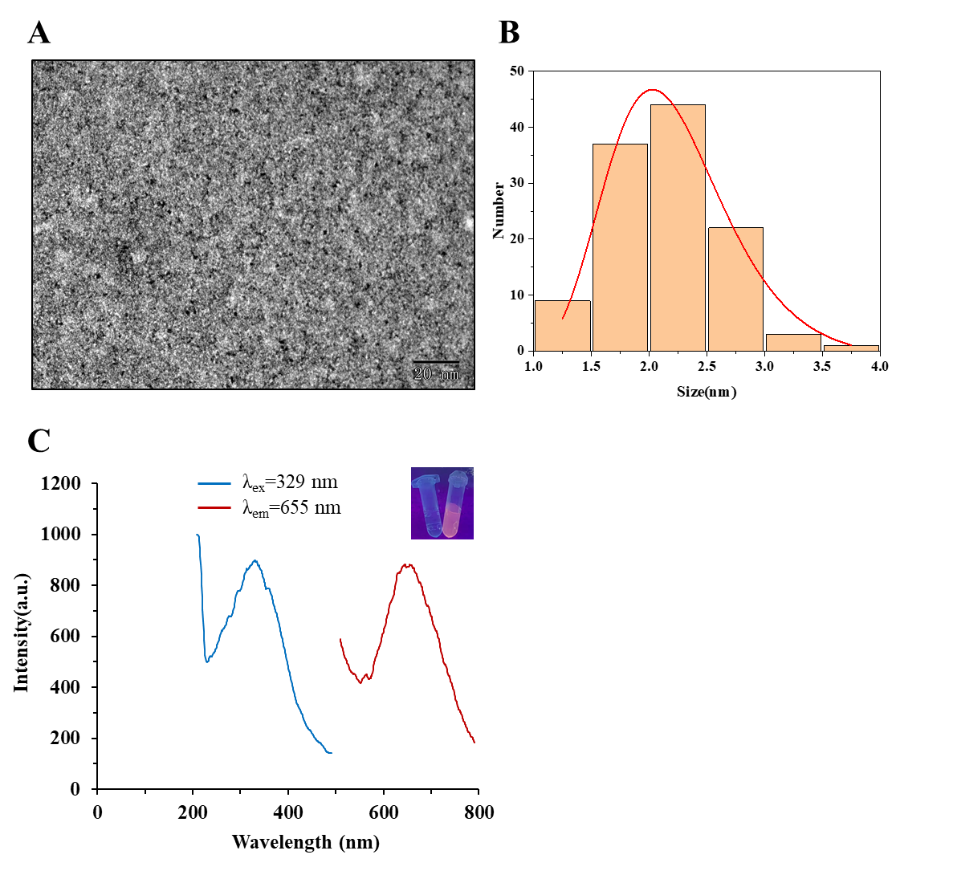
**

**Figure S3.** The successful synthesis of AuNC@PEI. A) TEM characterization of AuNC@PEI. B) Particle size distribution of AuNC@PEI in the dried calculated to TEM images. C) The fluorescence spectrum of AuNC@PEI.


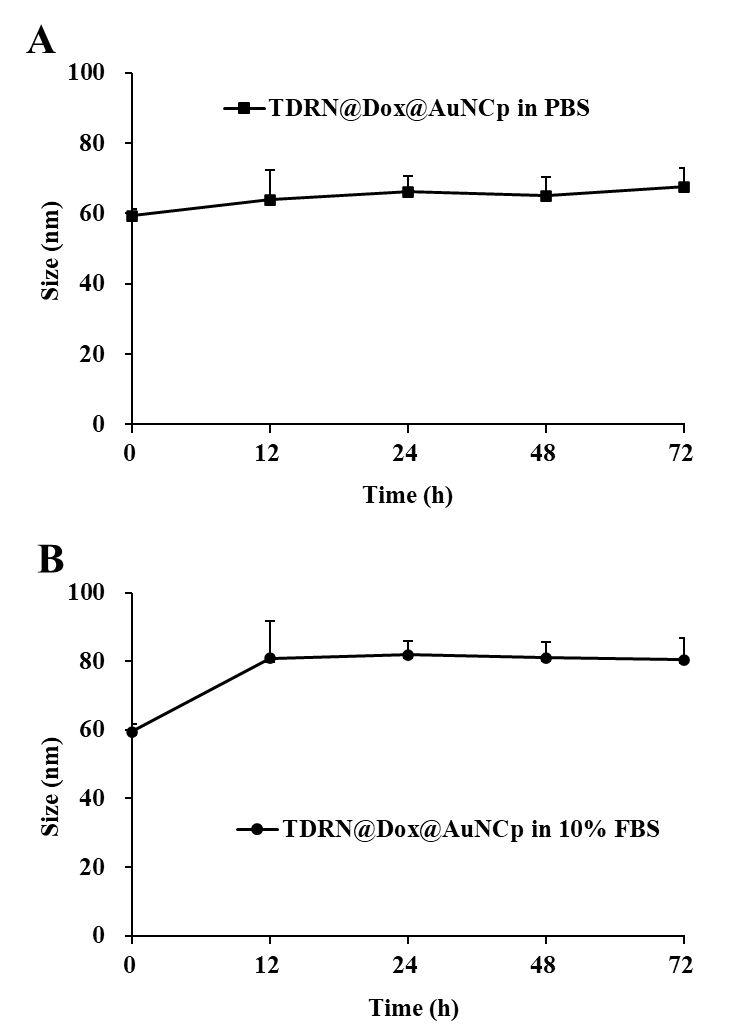
**Figure S4.** Stability analysis of TDRN@Dox@AuNC_p_ in PBS buffer (A) and 10%FBS solution (B) after storage for 0, 12, 24, 48 and 72 h, measured by DLS.


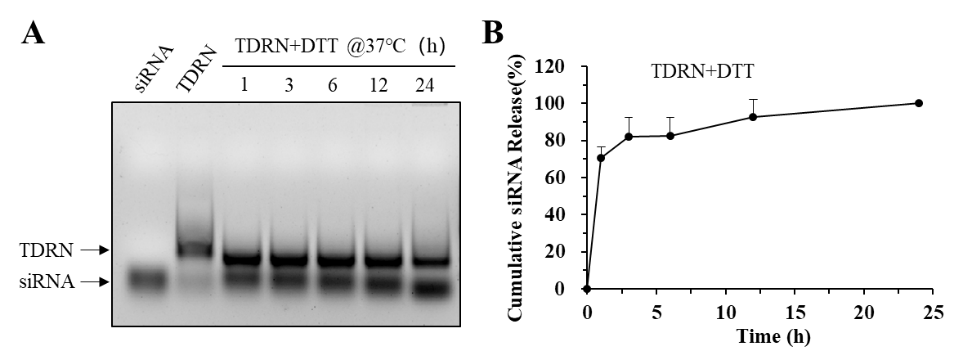


**Figure S5.** Analysis of DTT-responsive release properties of TDRN in solution. A) The TDRN was incubated with 5 mM DTT solution at 37℃ for predetermined time intervals and analyzed with 1.5% agarose gels. B) Percentages of cumulative siRNA release of the TDRN with 5 mM DTT solution were calculated using Image J software (n=3).

**
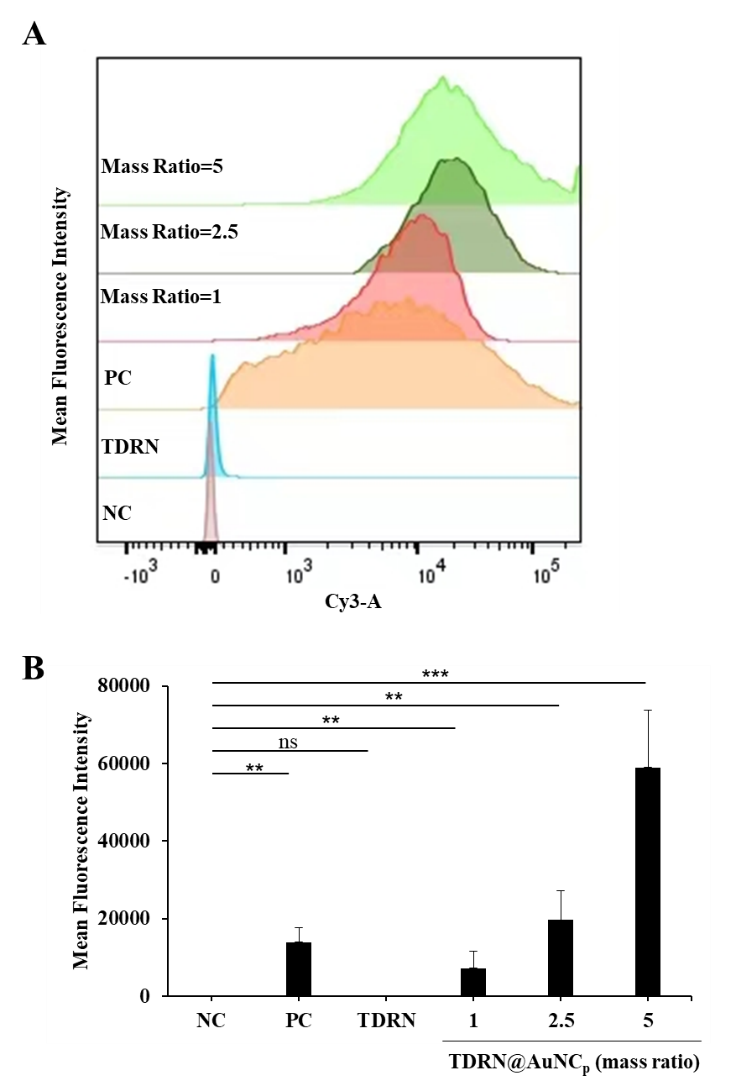
**

**Figure S6.** A) Cellular uptake of TDRN-Cy3@AuNC_p_ with different mass ratios in HeLa/ADR cells measured by flow cytometry analysis (A) and quantitative analysis of mean fluorescence intensity of Cy3 (B).


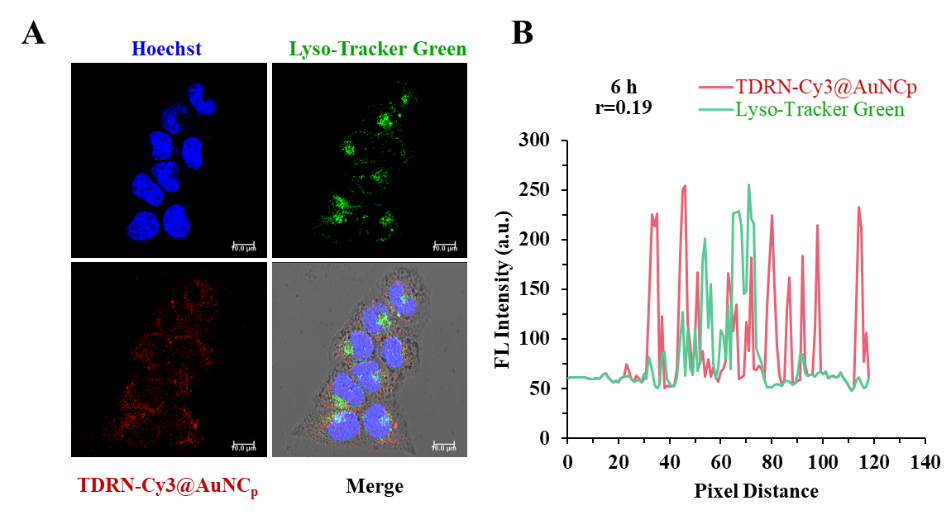


**Figure S7.** Lysosome escape of TDRN-Cy3@AuNC_p_ in HeLa/ADR cells for 6 h incubation using a confocal laser scanning microscope. A) CLSM images of TDRN-Cy3@AuNC_p_ in HeLa/ADR cells. B) The Pearson’s correlation coefficients between the green signals from Lyso-Tracker and red signals from TDRN-Cy3@AuNC_p_. Scale bar = 10 μm.

**
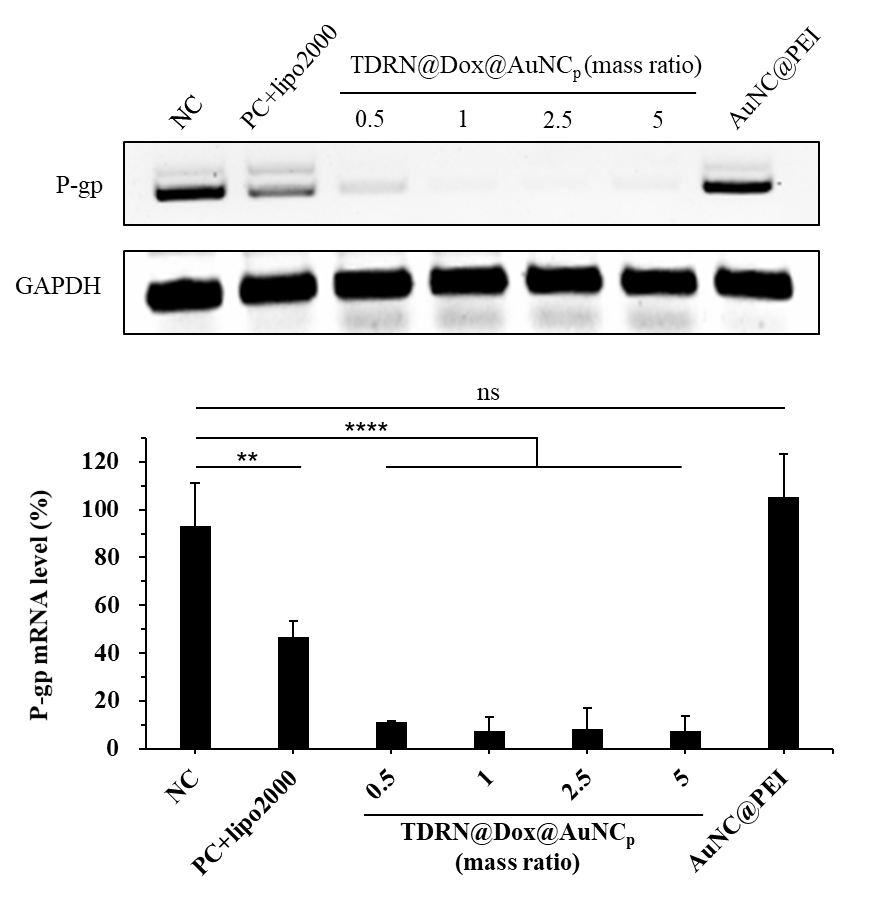
Figure S8**. Gene silencing efficiency of TDRN@Dox@AuNC_p_ in HeLa/ADR cells verified via RT-PCR treatment for 48 h. The image of agarose gels of RT-PCR demonstrated the gene silencing of TDRN@Dox@AuNC_p_. The gray-scale analysis of agarose gels using Image J software (n=3).

**
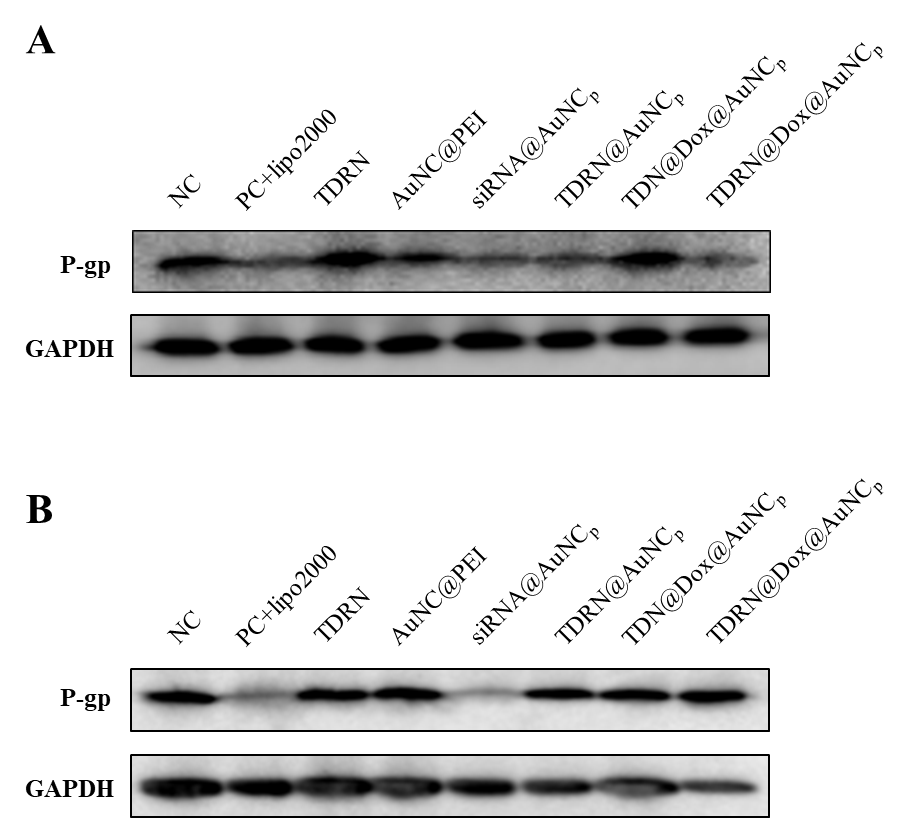
**

**Figure S9.** Western blot analysis of protein expression levels of P-gp and GAPDH with the TDRN@AuNC_p_, TDN@Dox@AuNC_p_ and TDRN@Dox@AuNC_p_ treatment for 48 h in GSH-responsive HeLa/ADR cells (A) and GSH-nonresponsive normal HUVEC cells (B).

**
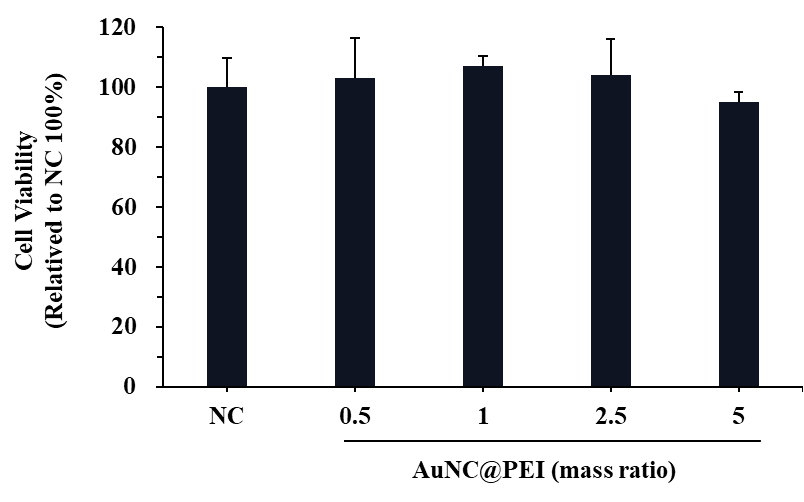
Figure S10.** The cytotoxicity of HeLa/ADR cells treated with AuNC@PEI with different mass ratios measured by MTT assay.

**
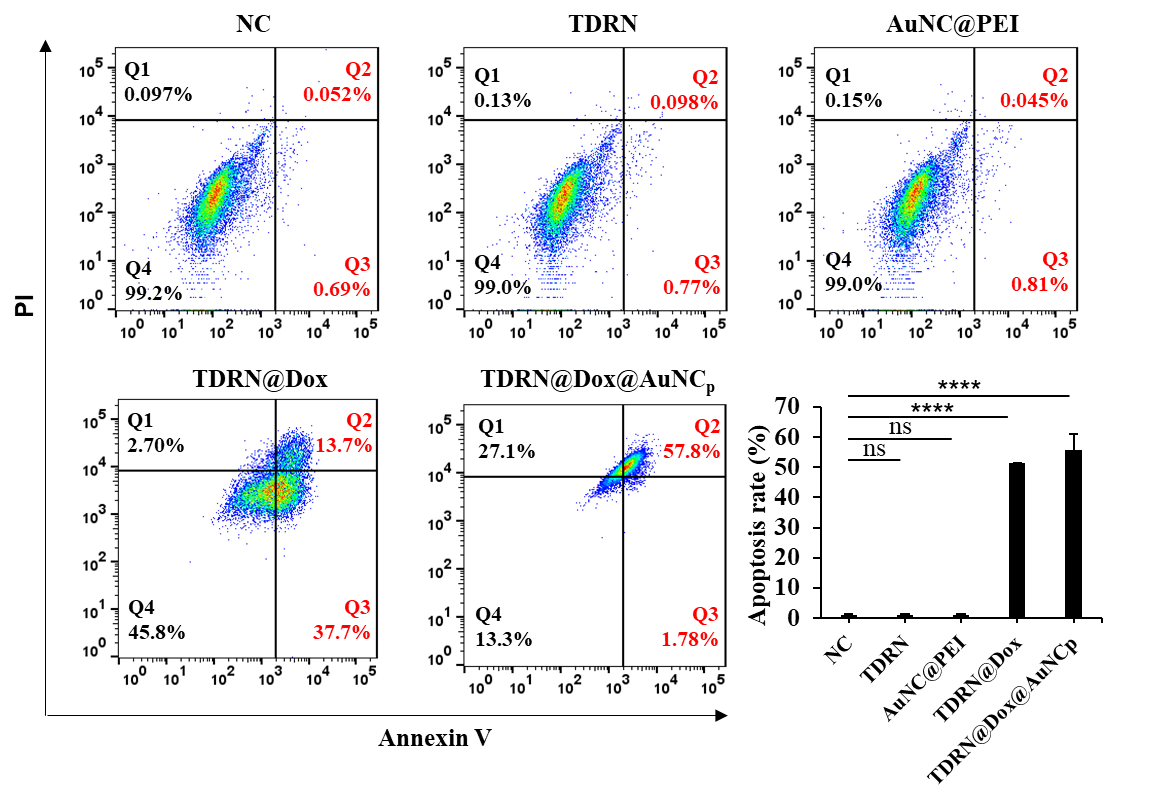
**

**Figure S11.** Cell apoptosis analysis in HeLa cells treated with TDRN, AuNC@PEI, TDRN@Dox, and TDRN@Dox@AuNC_p_ determined by flow cytometry and the apoptosis rates of HeLa cells were calculated using FlowJo software.

**
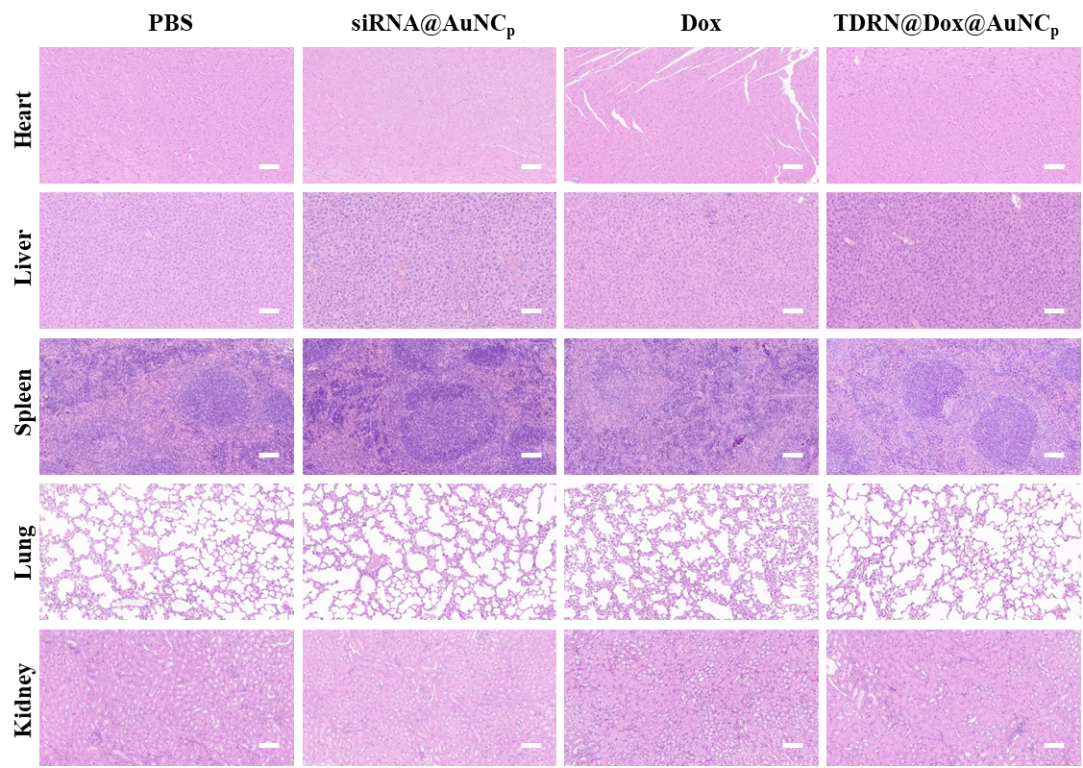
Figure S12.** H&E staining of heart, liver, spleen, lung and kidney on day 16 for all treatment groups. Scale bar = 100 μm.

**ESI-MS of oligonucleotides for TDRN**


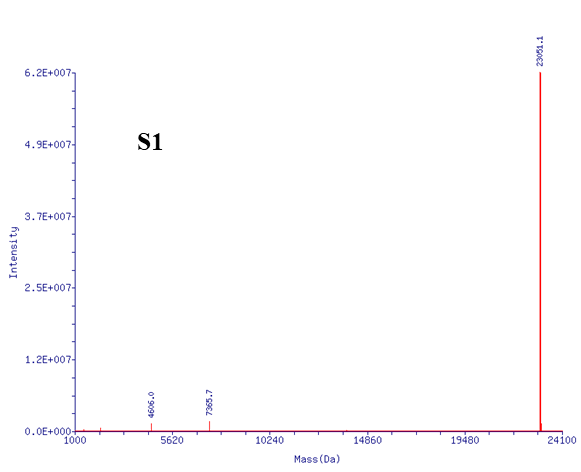


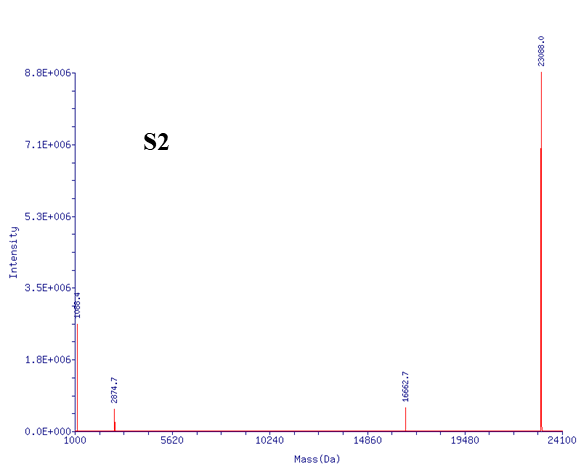

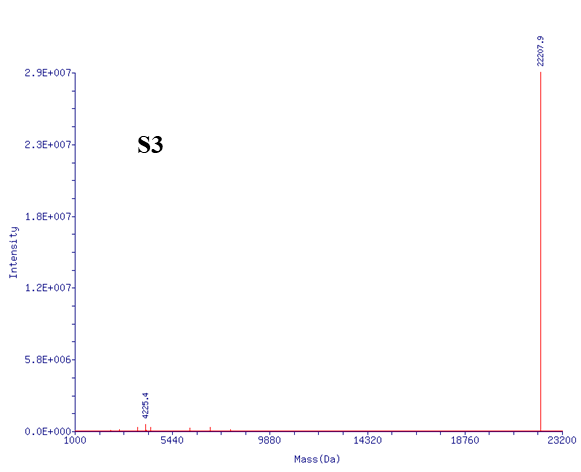


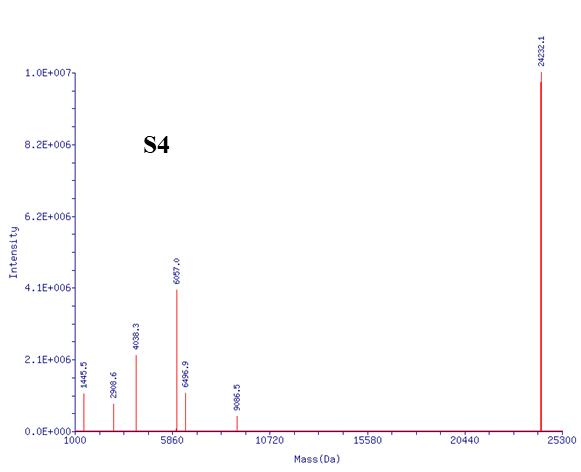


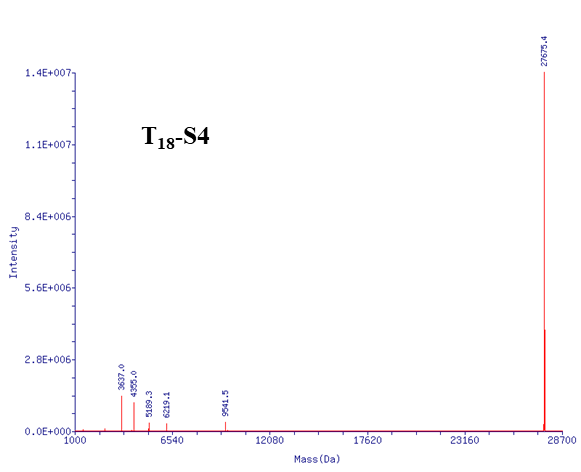


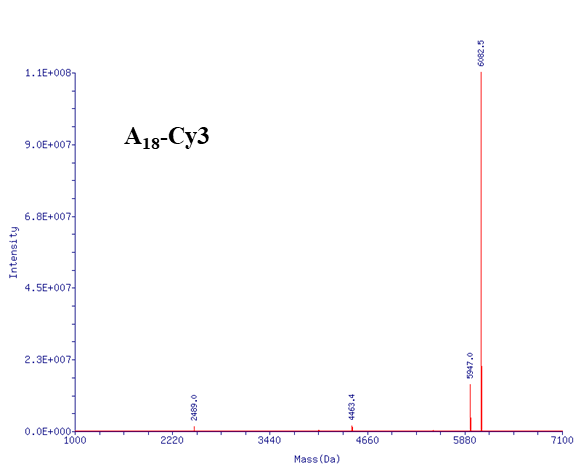

Supplement: Supplementary file 1 — Supporting Information [file ADVS-11-2404112-s001.docx]
